# Educating physicians in the era of genomic medicine

**DOI:** 10.1186/gm564

**Published:** 2014-06-27

**Authors:** Michael F Murray

**Affiliations:** 1 Director of Clinical Genomics, Genomic Medicine Institute, Geisinger Health System, 100 N. Academy Ave, MC 26-20, Danville, PA 17822, USA

## Abstract

Michael F Murray, MD, of Geisinger Health System in Pennsylvania discusses how to meet the need for better training for physicians in genomics as it moves further into the clinic.

## Introduction

**Figure 1:**
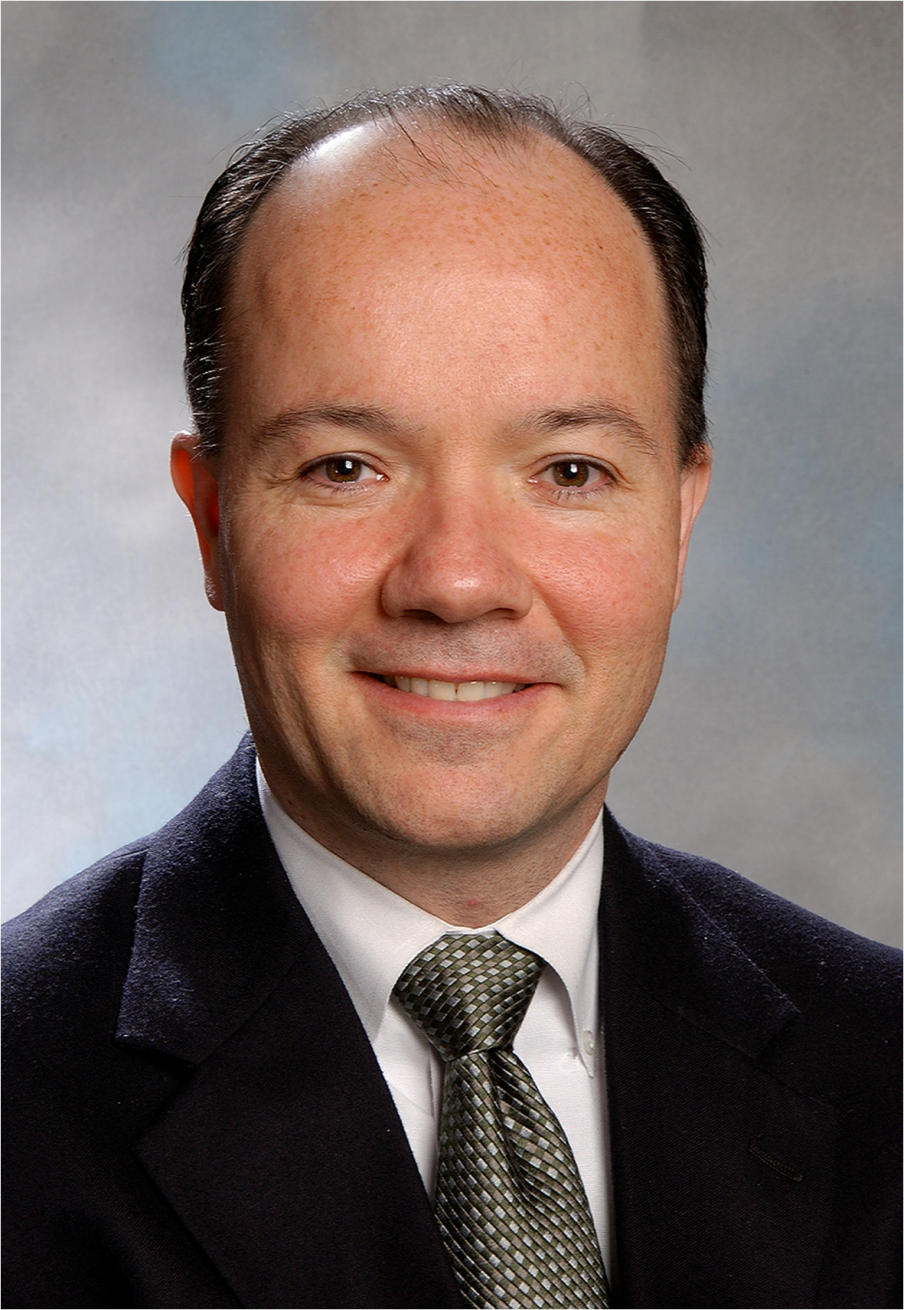
Michael F Murray

Michael F Murray, MD [Figure 1], is Director of Clinical Genomics at the Genomic Medicine Institute of Geisinger Health System in Danville, Pennsylvania. As part of his involvement in projects such as MedSeq, he is exploring innovative ways of addressing the increasing need for physicians to be educated about genomics.

## Is the current standard of genomics education for physicians adequate?

Unfortunately, there is no standardized approach in the United States to genomics education for physicians. Despite numerous calls for more genomics education, stretching out for over a decade, most of us who are involved in these efforts feel that the physician workforce is currently unprepared for any large-scale application of genomic medicine [1,2].

In recent years a number of medical schools have increased the amount of genomics that they incorporate into the curriculum, so it is likely that more recent graduates have a more extensive educational exposure to genomics. The ‘Genes to Society’ curriculum launched in 2009 at Johns Hopkins University in Baltimore, Maryland, in particular stands out in this regard [3]. However, there is still a broad range of exposure to genomics education even in recent medical school graduates, depending on their medical school.

For residents and fellows in graduate medical education (GME) training, the educational programs also vary widely. There are of course specific GME training programs in clinical and laboratory genetics and genomics; however, these account for a very small fraction of trainees.

In the postgraduate medical education world - of practicing physicians - there is currently no requirement for non-genetic professionals to have any specific knowledge or competencies in genomics, and so genomics is at a competitive disadvantage for the attention of professionals who are deciding among many important clinical education topics.

## Does the level of genomics education differ among clinical specialties?

The primary driver of educational topics for specialties is the frequency at which an area of knowledge comes up in clinical practice of that specialty. This then indirectly drives the incorporation of education topics into formal curriculums and standardized examinations. With that in mind, specialists in clinical genetics have that as their expertise focus and so they have the most education on the application of genomics in patient care.

Other than geneticists I think that pediatrics, obstetrics, and pathology are likely the three primary specialty areas with the most genomics training. Among subspecialists, oncologists have the most applications in their clinical work for genetics and genomics, and would therefore likely be the best-educated professional group.

## Do you think that physicians are aware of the range of genomic diagnostic tests that are already available?

In general, once a provider starts to use a laboratory test they usually get to the point pretty quickly where they have a working knowledge of its performance characteristics. However, with genetics and genomics a significant percentage of providers have never ordered testing for a patient. In surveys of physicians about their awareness of genetic and genomic testing options they generally admit that they do not have a working understanding of the testing menu available or the specific indications for the tests. For instance, there seems to be general confusion amongst clinicians around the distinction between genomic sequencing and array-based genotyping.

In the area of testing options I suspect that we will ultimately move to a whole-genome approach for all, which will of course make some of the debate about test selection obsolete. It is not clear at this point whether or not improved familiarity with available genomic tests would lead to significant improvements in patient care across the healthcare system. However, there is definitely anecdotal evidence of cases where the diagnostic use of genomics could have improved patient management.

## How will genomics education for physicians need to change, given the increasing impact of genomics on clinical practice?

Since this is new territory for most providers, and it competes with many other topics for the limited professional time that physicians can set aside for education, strategies for genomics education have to be integrated with other priorities in order to really gain significant uptake. So offering continuing medical education (CME) credit for participation in educational activities is essential since it then aligns with a necessity for professional certification.

Other ways to integrate genomics training include the development of point-of-care educational opportunities that are readily available to providers at the moment when they are most highly motivated to learn - for instance, offering educational opportunities linked to the electronic health record about gene-drug pairs in pharmacogenomics when the drug is prescribed. Some of that kind of point-of-care pharmacogenomics decision support and education is being piloted within the Electronic Medical Records and Genomics (eMERGE) network [4].

Another way to align educational efforts with professional requirements is to work towards having all of the American Board of Medical Specialties (ABMS) certifying boards offer maintenance of certification (MOC) credit around activities that focus on genomics and to have those boards incorporate genomics knowledge questions into board certification and re-certification exams.

## What options are being explored to change or expand genomics education for physicians?

The National Human Genome Research Institute (NHGRI) has initiated an effort to engage professional societies in genomics education. The Inter-Society Coordinating Committee for Practitioner Education in Genomics (ISCC) [5] recognizes that many practitioners look to their trusted professional organizations to understand educational priorities and to find educational instruments. This effort has great potential for improving targeted education within different provider groups [6].

Two other efforts that I have been involved in will use targeted educational instruments delivered to physicians involved in genomic results management in two different settings. The first is the MedSeq project, in which primary care providers at Brigham and Women’s Hospital in Boston, Massachusetts, are delivering genomic results to their patients after going through a ‘just-in-time’ 6.5-hour genomics training course [7]. The second is a project at Geisinger Health System in which genomic results from the American College of Medical Genetics (ACMG) incidental findings list are delivered to patients. Here, primary care providers have the option of point-of-care education just prior to that result delivery.

## Can you give us any initial insights from these projects as to how successful these different strategies are likely to be?

The ISCC has generated a framework to support different provider groups as they define their own specific competencies in genomics [8]. We hope that this will allow the workload of creating educational efforts to be spread among a broad range of experts, and ultimately allow motivated physician learners to find the most relevant material for their practice.

We hope to continue to improve upon the MedSeq and Geisinger Health System educational efforts and then ultimately make the best elements of them available to learners outside of these projects.

## How do you think that physicians at different stages of their career will cope with these changes?

Practicing clinicians are highly strategic and very effective learners. I like to cite the example of infectious disease specialists who very rapidly acquired new and evolving knowledge around the management of HIV infection in the 1990s and into this century because it was essential to providing good care for their patients. The same is true when we look at the current changes in the use of tumor genomics, and the rapid acquisition of genomics knowledge by oncologists as the evidence for improved outcomes through this approach is rapidly mounting.

I think that providers at all career stages will easily cope with the task of understanding new information that they believe is useful for patient care. One of the reasons for pushback from many providers about applying genotyping to clinical disease risk prediction for individual patients, as offered by some direct-to-consumer companies, is that there remains very limited evidence for the clinical utility of that approach. If an evidence base develops, then I think that providers will seek to become educated on how to employ these strategies.

## Is expanded education sufficient, or will physicians need more continual support as the impact of genomics increases?

I think that we will all need continual education in this area as new knowledge is generated from research. As more clinically applicable genomics knowledge becomes available there will be more interest and need for educational support.

In addition to educational courses, old-fashioned tools like textbooks will remain critical resources for genomic medicine implementation; and of course the electronic versions are so much easier to carry around. There is a great textbook called ‘Genomic and Personalized Medicine’ by Geoff Ginsburg and Hunt Willard that is now in its second edition [9]. And a group of us just came out with a book called ‘Clinical Genomics: Practical Applications for Adult Patient Care’ [10]. Both of these books really organize the genomics knowledge base in ways that are familiar and useful to practicing clinicians, and draw on the rapidly expanding knowledge base, the majority of which has developed in just the last 10 years. Clinicians will need these kinds of reference tools, ideally electronically linked into their busy workflow.

Of course genomics will not stand alone but will be integrated with other new knowledge domains such as proteomics and metabolomics, which will also require an extensive amount of educational training to providers in order for them to be applied to patient care.

## As well as the need to interpret more and more complex genomic information, is there also a need to educate physicians differently in terms of their relationship with patients?

Yes there are knowledge areas other than genomic science that need to be included in genomic education. The wisdom of the inclusion of ethical, legal and social implication perspectives into genomics research needs to be extended into provider clinical education about the use of genomics.
